# Butyrate Mitigates Weanling Piglets From Lipopolysaccharide-Induced Colitis by Regulating Microbiota and Energy Metabolism of the Gut–Liver Axis

**DOI:** 10.3389/fmicb.2020.588666

**Published:** 2020-12-08

**Authors:** Yunsheng Han, Qingyu Zhao, Chaohua Tang, Ying Li, Kai Zhang, Fadi Li, Junmin Zhang

**Affiliations:** ^1^State Key Laboratory of Grassland Agro-Ecosystems, Key Laboratory of Grassland Livestock Industry Innovation, Ministry of Agriculture and Rural Affairs, College of Pastoral Agriculture Science and Technology, Lanzhou University, Lanzhou, China; ^2^State Key Laboratory of Animal Nutrition, Institute of Animal Sciences of Chinese Academy of Agricultural Sciences, Beijing, China; ^3^Scientific Observing and Experiment Station of Animal Genetic Resources and Nutrition in North China of Ministry of Agriculture and Rural Affairs, Institute of Animal Science of Chinese Academy of Agricultural Sciences, Beijing, China

**Keywords:** colitis, energy metabolism, gut–liver axis, microbiota, piglet, protected butyrate

## Abstract

Inflammatory bowel disorder is accompanied by the destruction of immunity homeostasis, gut microbiota perturbation, and chronic inflammatory liver diseases. Butyrate is known as a primary energy source for colonocytes and functional substances for mitigating pathological features of colitis. However, it is still unclear whether butyrate alleviates colitis progression by regulation of microbiota and metabolism in the gut–liver axis. In the present study, we aimed to determine the role of microbiota and metabolism of the gut–liver axis in ameliorating lipopolysaccharide (LPS)-induced colitis in piglets using protected butyrate administration. Eighteen crossbred male piglets were weaned at 30 days old and were randomly allocated to three treatments, with CON (basal diet), LPS (basal diet + LPS), and BT-LPS (basal diet + 3.0 g/kg protected butyrate + LPS). On days 19 and 21, piglets in the LPS and BT-LPS groups were intraperitoneally challenged with LPS at 100 μg/kg body weight. Butyrate administration significantly decreased LPS-induced rise in the clinical score of piglets and colonic histological scores and reduced the susceptibility to LPS-induced severe inflammatory response by decreasing proinflammatory (IL-1β, IL-6, IL-8, and TNF-α) cytokines. Butyrate supplementation accelerated the prevalence of *Faecalibacterium* and *Lactobacillus* by enhancing the tricarboxylic acid (TCA) cycle of colonocytes. Dietary supplementation with protected butyrate significantly targeted increased concentrations of butyric acid in the colon and portal venous circulation, and enhanced the TCA cycle in the gut–liver axis by mobilizing amino acid and vitamin B group as a coenzyme. Meanwhile, during this progress, LPS increased fatty acid synthesis that was reversed by butyrate treatment, which was reflected by decreased acylcarnitines. Butyrate-reshaped colonic microbial community and metabolism in the gut–liver axis contributed to morphology integrity and immunity homeostasis by promoting anti-inflammatory (IL-10 and TGF-β) cytokines and suppressing inflammatory mediator hypoxia-inducible factor 1α and its downstream response elements cyclooxygenase 2 and inducible nitric oxide synthase. These results identified the pivotal role of colonic microbiota and metabolism in the gut–liver axis for alleviating inflammatory progression and possible therapeutic targets.

## Introduction

Inflammatory bowel disorders (IBDs), which include Crohn’s disease and ulcerative colitis, are worldwide diseases characterized by a chronic and recurrent inflammatory response and injury of the colon (Shi et al., 2016). Emerging data based on high-throughput sequencing and metabolomics methods link the pathogenesis of IBD and metabolic disorders to an aberrant gut microbiota composition (Koh et al., 2016). Therefore, some clinical applications of therapies have focused on exploring efficient therapies for IBD in a microbial manipulation-dependent method such as intervention therapy of antibiotics, probiotics, and prebiotics (Anna et al., 2011). However, its therapeutic efficacy is non-ideal. Butyrate, one of the short-chain fatty acids (SCFAs), is derived from microbial fermentation of dietary fibers and appears to have multiple beneficial effects on host physiology. Gut microbiome analysis has demonstrated a significant decrease in the number of butyrate-producing bacteria in the colons of patients with IBD (Frank et al., 2007). Direct application of butyrate by colonic irrigation alleviates inflammation during IBD (Hamer et al., 2008; Postler and Ghosh, 2017). Butyrate has consequently received much attention for its effects on gut health.

The liver features a direct anatomical link to the gut by portal venous circulation, thus continually exposed to bacterial products such as SCFAs, in which the connection is considered as the “gut–liver axis.” The gut–liver axis is an interconnected system that functions to process gut-derived products, regulate metabolism, and maintain immunity homeostasis (MacDonald and Monteleone, 2005). A common feature of IBD is the influx of disruptive immune cells *via* the portal circulatory system, indicating the destruction of immunity homeostasis (Podolsky, 1991). This resulted in 80% of patients with IBD having a concurrent autoimmune liver disease (Loftus, 2005). SCFAs, whose function ranges from being an energy source to being a key mediator for immune cell functions, could increase liver metabolism, enhance gut barrier function, and moderate IBD-associated symptoms (Singh et al., 2014; Trapecar et al., 2020). Thereby, SCFAs might play an important role in IBD therapy through regulation of the gut–liver axis.

The microbiome can be considered as one of the drivers to influence the gut–liver axis, and can then influence immune function. A healthy microbial community coordinates the balance of effector and regulatory immune cells, as well as anti-inflammatory and pro-inflammatory effects *via* its metabolites. This was to some extent demonstrated by previous studies that antibiotic-treated and germ-free mice are more susceptible to dextran sulfate sodium (DSS)-induced colitis (Rakoff-Nahoum et al., 2004; Maslowski et al., 2009). Although recent evidence has depicted in some detail how the immune system maintained homeostasis by shaping the microbial community to be beneficial (Foster et al., 2017), host cell types and corresponding mechanisms in balancing microbiota and the immune system are still little known. The colonocytes seemed to have two opposing colonocyte phenotypes and metabolic polarization. In healthy gut, butyrate-activated PPAR-γ signaling increased differentiated colonocytes by polarizing its intracellular metabolism toward mitochondrial β-oxidation of fatty acids (Litvak et al., 2018), while colonocyte phenotype was changed by reprogramming its metabolism toward anaerobic glycolysis when pro-inflammatory signals were present (Byndloss and Bäumler, 2018). This evidence highlights the central role of the gut–liver axis between microbiota and immunity homeostasis.

Lipopolysaccharide (LPS) is derived from the outer membrane of Gram-negative bacteria, which participates in chronic inflammation and aggravates the progression of disease (Gioannini and Weiss, 2007; Miyake, 2007). Toll-like receptor (TLR) 4, as a member of the natural immune pattern recognition receptors family, contributes to innate immunity through recognition and combination of various pathogen-associated molecular patterns. The interaction of agonistic LPS with the host MD-2/TLR4 complex triggers inflammatory signaling cascades *via* myeloid differentiation factor 88 (MyD88)-dependent and/or MyD88-independent pathways (Tanimura et al., 2008; Motshwene et al., 2009), leading to the nuclear translocation of the transcription factor NF-κB, subsequently resulting in the production of cytokines, such as TNF-α, IL-1β, and other proinflammatory mediators promoting an inflammatory response (Cochet and Peri, 2017; Chen et al., 2020). In this process, it is commonly accompanied by inflammatory cell infiltration and epithelial damage (Chen et al., 2018a; Geng et al., 2018; Fan et al., 2019). Because the mechanism is well-established, LPS-induced intestinal injury was taken as an inflammatory stress model *in vivo* (Liu et al., 2008; Fan et al., 2019), and the LPS-challenged IPEC-J2 epithelial monolayer was used as a model *in vitro* (Hu et al., 2020). In addition, previous studies utilized piglets challenged with LPS as an IBD model to study the therapeutic efficacy of fecal microbiota transplantation on epithelial injury (Geng et al., 2018).

Although we observed that butyrate benefited gut health, it is still largely unknown how the mechanism of protected butyrate alleviates colitis. In this study, we hypothesize that protected butyrate might reshape the gut microbiota, and alter metabolism in the gut–liver axis, subsequently contributing to immunity homeostasis and alleviating colitis. Therefore, this study employed the LPS-induced colitis model in piglets to explore further the mechanisms by which butyrate intervention reduces colitis through regulating colonic microbiota and energy metabolism in the gut–liver axis. The results provide insights into the potential mechanisms underlying the modulatory effect of butyrate during its applications for the treatment of IBD.

## Materials and Methods

### Ethics Statement

This study was carried out in accordance with the recommendations of “Guidelines on Welfare and Ethical Review for Laboratory Animals” (GB/T 35892-2018) approved by the Institutional Animal Care and Use Committee of the Institute of Animal Science of the Chinese Academy of Agricultural Sciences (IAS2019–66, Beijing, China).

### Experimental Design and Sample Harvesting

Eighteen crossbred male piglets (Duroc × Landrace × Yorkshire, weaned at 30 ± 2 days) with an average initial body weight (BW, 9.10 ± 0.15 kg) were selected and randomly allocated to three groups. Each group consisted of six replicates (pens), with one piglet per pen. A non-medicated corn-soybean basal diet in mashed form was formulated to meet the nutrient requirements of the National Research Council (2012) for 11–20-kg pigs (Table 1). The three groups included basal diet and challenging with sterile saline (CON, *n* = 6), basal diet and challenging with LPS (LPS, *n* = 6), and basal diet with a single dose of 3 kg protected sodium butyrate per ton of feed and challenging with LPS (BT-LPS, *n* = 6). On days 19 and 21 during the 21-day feeding trial, piglets in the LPS and BT-LPS groups were challenged intraperitoneally with LPS (*Escherichia coli* serotype 055:B5; Sigma-Aldrich, St. Louis, MO, United States) at 100 μg/kg BW dissolved in sterile saline (Fan et al., 2019), while piglets in the CON group were accordingly given sterile saline in an equivalent dosage. All piglets were housed in an environmentally controlled room with a hard plastic, fully slotted floor. The adjacent pens were separated by a closed baffle. Piglets were given *ad libitum* access to feed and water. The room temperature was controlled at 28–30°C. Protected butyrate supplementation was provided in the form of Gustor BP70 (Norel S.A., Madrid, Spain) encapsulated using intelligent microencapsulation technology, a formulation of partially protected sodium butyrate composed of 70% sodium butyrate and 30% fat. The dosage was chosen according to previous studies with the same protected butyrate (Casanova-Higes et al., 2017; Sanggun et al., 2018).

**Table 1:** Ingredients and chemical composition of experimental diets.

Ingredient (%)	Content
Extruded corn	55.00
Soybean meal	11.30
Extruded soybean	10.00
Fish meal	5.00
Soybean protein concentrate	4.00
Whey powder	8.00
Sucrose	2.00
Soy oil	1.50
Dicalcium phosphate	1.00
Limestone	0.50
Salt	0.20
Chromium oxide	0.25
l-Lysine-HCl	0.30
dl-Methionine	0.20
l-Threonine	0.15
l-Tryptophan	0.10
Premix^†^	0.50
**Nutrient composition^‡^**	
Digestible energy (MJ/kg)	14.50
Crude protein (%)	19.10
Calcium (%)	0.82
Total phosphorus (%)	0.72
Digestible phosphorus (%)	0.49
SID lysine (%)	1.23
SID methionine (%)	0.36
SID threonine (%)	0.74
SID tryptophan (%)	0.20

† Provided per kg of diet: vitamin A, 2200 IU; vitamin D_3_, 220 IU; vitamin E, 11 IU; vitamin K_3_, 0.5 mg; vitamin B_12_, 0.015 mg; riboflavin, 4 mg; niacin, 30 mg; pantothenic acid, 10 mg; choline chloride, 400 mg; folic acid, 0.3 mg; thiamine, 1.5 mg; vitamin B_6_, 3 mg; biotin, 0.1 mg; zinc, 100 mg; manganese, 4 mg; iron, 84 mg; copper, 6 mg; iodine, 0.14 mg; and selenium, 0.35 mg.

‡ Nutrient levels are calculated.

Taking each piglet as one unit, BW was recorded at the beginning of the experiment and on days 14 and 21 to calculate the average daily gain (ADG) during days 15–21 and 1–21. Piglets were monitored from day 15 to 21 with a clinical severity score that is varied from 0 (normal) to 15, as described by Li et al. (2012). On day 21, after fasting for 12 h, whole blood samples from all the piglets were collected into procoagulant vacuum tubes *via* jugular vein puncture at 4 h after LPS challenge. Serum samples were obtained by centrifugation for 10 min (3,000 *g*, 4°C) and were stored at −20°C until further analysis of cytokines. Following blood collection (at 4 h after LPS challenge), piglets were slaughtered 5 min after injection of the anesthetics. Colonic samples were harvested and fixed in 4% formalin and stored at 4°C. Fresh colonic contents, tissue, and mucosa from the remaining colon segments were obtained as described previously (Wang et al., 2008), and immediately frozen in liquid nitrogen and stored at −80°C for further analysis of SCFAs and bacterial genomic DNA, immune cytokines, and target energy metabolites. Liver was obtained as described previously (Wang et al., 2008; Huang et al., 2020), and then stored at −80°C after being rapidly placed in liquid nitrogen for further analysis of metabolites.

### Colonic Morphology, Histological Scores, and SCFA Profile Analysis

Tissues were processed in Historesin (Leica Microsystems, South San Francisco, CA, United States) and 4-μm sections prepared for staining with hematoxylin and eosin. Slides were analyzed using a microscope. Samples were analyzed blindly with histological scores system from 0 to 30 for each parameter, as described by Fachi et al. (2019). SCFAs in the colon were measured using Agilent 6890N GC (Palo Alto, CA, United States) according to a previous study (Zhang et al., 2018b). SCFAs in serum were measured using liquid chromatography-tandem mass spectrometry, according to a previous study (Han et al., 2015).

### Biochemical Analysis

Serum proinflammatory cytokines (IL-1β, IL-6, IL-8, IL-12, and IL-17), tumor necrosis factor-α (TNF-α), nuclear factor κB (NF-κB), and anti-inflammatory cytokines (IL-10) and transcription growth factor β (TGF-β) were measured using an ELISA assay kit (Shanghai Enzyme-linked Biotechnology Co., Ltd., China) following the manufacturer’s instruction. Regarding colonic immune indexes, 100 mg colon tissue was mixed with 1 ml precooled sterile saline, ground in ice using a superfine homogenizer vortexed for 30 s, and centrifuged for 15 min (14,000 *g*, 4°C). The supernatant was transferred to a new centrifuge tube and was used to measure immune indexes with an ELISA assay kit (Shanghai Enzyme-linked Biotechnology Co., Ltd., China). The indexes included pro-inflammatory associated cytokines (IL-1β, IL-6, IL-8, IL-12, IL-17, TNF-α, and NF-κB); anti-inflammatory cytokines (IL-10 and TGF-β); and hypoxia-inducible factor 1α (HIF1α), cyclooxygenase 2 (COX-2), and inducible nitric oxide synthase (iNOS). The obtained results were further normalized with the tissue protein, which was measured with a BCA commercial kit (Thermo Fisher Scientific, Waltham, MA, United States) according to the manufacturer’s protocols.

### Colonic Microbiota Analysis

Bacterial genomic DNA was extracted from each colonic chyme sample (Qiagen DNA stool Mini Kit, Germany). DNA was quantified with a NanoDrop 2000 spectrophotometer (Thermo Scientific) and was further assessed by running on 1% agarose gels. The V3–V4 hypervariable region of 16S rRNA genes was amplified using specific primer pairs (forward 5'-ACTCCTACGGGAGGCAGCA-3' and reverse 5'-GGACTACHVGGGTWTCTAAT-3') with barcodes to construct the sequencing libraries (TruSeq® DNA PCR-Free Sample Prep Kit, Illumina, San Diego, CA, United States). The qualified DNA libraries were loaded in a NovaSeq platform with 2 × 250 bp paired-end sequencing. The paired-end reads were obtained and merged using FLASH software (V1.2.7, http://ccb.jhu.edu/software/FLASH/). A total of 1,440,362 effective sequences (from 1,507,327 raw reads) were obtained by sequence-filtering in QIIME (V1.9.1, http://qiime.org/scripts/split_libraries_fastq.html) and chimera-removing in UCHIME algorithm.^1^ Operational taxonomic units (OTUs) with a 97% identity were gathered with Uparse (ver. 7.1, http://drive5.com/uparse/). Taxonomic annotation was performed using the Mothur algorithm (70% confidence) with the Silva Database.^2^ Alpha-diversity indices were presented using the Chao 1 estimator^3^ and Shannon estimator.^4^ Beta-diversity was visualized using a nonmetric multidimensional scaling analysis (NMDS) plot and a principal coordinate analysis (PCoA) plot with a weighted Unifrac index. The bacterial biomarkers within groups were explored using the linear discriminant analysis effect size [LEfSe, linear discriminant analysis (LDA) > 4] and were presented from phylum to genus levels. Phylogenetic investigation of communities by reconstruction of unobserved states (PICRUSt) was used to estimate metagenome functional content. A spectrum of bacterial functionalities was predicted using the PICRUSt analysis based on different Kyoto Encyclopedia of Genes and Genomes (KEGG) levels (Langille et al., 2013). The relative percentages of predictive pathways on KEGG level II were presented as a heatmap plot with the normalized analysis.

### Targeted Metabolomics Analysis

Metabolite extraction and mass spectrum (MS)-based metabolomics analysis were performed as previously reported (Rahman et al., 2018). High performance liquid chromatography (HPLC)-grade methanol (700 μl, 80% v/v) was cooled to −80°C and added to a 1.5-ml tube with 70 mg homogenate of colonic mucosa and liver, incubated at −80°C for 2 h, and centrifuged for 20 min (20,000 *g*, 4°C). The supernatant was transferred to another 1.5-ml centrifuge tube and evaporated to dryness. The residues were reconstituted in 80% methanol for further analysis. Targeted metabolomic analysis was performed using TSQ Quantiva (Thermo Fisher Scientific) in a positive-negative ion switching mode. Reverse-phase chromatography (C18 column) was carried out using 10 mM tributylamine, 15 mM acetate in water and 100% methanol as mobile phases A and B, respectively. A 25 min gradient from 5 to 90% of mobile phase B was used. The resolutions for Q1 and Q3 were both 0.7 FWHM. The source voltage was 3,500 V for positive ion mode and 2,500 V for negative ion mode. The source parameters were as follows: spray voltage, 3,000 V; capillary temperature, 320°C; heater temperature, 300°C; auxiliary gas flow rate, 10 Arb; and sheath gas flow rate, 35 Arb. Metabolite identification was performed using Tracefinder 3.2 (Thermo Fisher Scientific) with a home-built database (Che et al., 2018).

### Statistical Analysis

Data were analyzed using a one-way ANOVA in SPSS 22.0 (IBM Corp., Armonk, NY, United States). The Tukey’s HSD test was employed to test the differences in microbial diversity. LEfSe analysis and Metastat analysis were performed to test significant differences in the relative abundance of the microbiota. The differences among treatment means for the clinical and histological scores, immune indexes, and hydrophilic metabolites were analyzed using Duncan’s multiple-range test and least significant difference (LSD) *post hoc* tests. Partial least squares–discriminant analysis (PLS–DA) and pathway analysis were conducted using Metaboanalyst 4.0 online (Jasmine et al., 2018). The correlation between differential gut microbiota or metabolites and immune parameters, and corresponding *p*-values were estimated using Spearman’s correlation analysis with the gplots and psych packages, respectively, in R (Version 3.5.3). Statistical results are shown as mean ± SEM, values of *p* < 0.05 (^*^) were considered as statistically significant, and values of *p* < 0.01 (^**^) were defined as extremely significant.

## Results

### Butyrate Improved Piglet Clinical Scores, Protected Colonic Morphology, and Mitigated Colitis Caused by LPS

**Figure 1:**
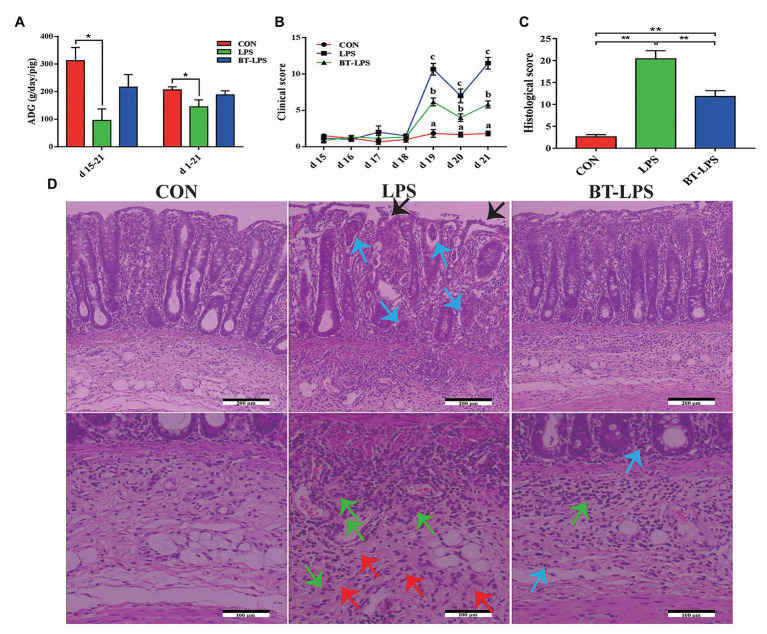
Dietary supplementation with butyrate improved clinical scores and protected colonic morphology of weaned piglets caused by lipopolysaccharide (LPS). **(A)** Average daily gain (ADG) of piglets. **(B)** Clinical scores of piglets based on an evaluation system of 0–15 scores. **(C)** Colonic histological scores on day 21 based on an evaluation system of 0–30 scores. **(D)** Representative colon histological sections of LPS-challenged and butyrate-treated piglets. Arrows in the sections indicate major histopathological differences between treatments: epithelial damage (black arrow), granulocyte inflammation (blue arrow), monocyte inflammation (green arrow), and submucosa edema (red arrow). All values are expressed as the mean ± SEM (*n* = 6). ^*^p < 0.05 and ^**^p < 0.01. a–c in the figure are used to mark the significant differences among treatments, indicating *p* < 0.05.

Compared with the CON group, LPS challenge significantly decreased piglets’ ADG during days 15–21 and 1–21, and increased clinical scores during days 19–21 (*p* < 0.05). In comparison with the LPS group, butyrate treatment significantly decreased the clinical scores (*p* < 0.05), which were significantly higher than those in the CON group (*p* < 0.05; Figures 1A,B). A histological examination of the colon indicated that the LPS challenge caused epithelial damage (black arrow; Figure 1D). Severe edema in the submucosa (red arrow) and extensive infiltration of inflammatory cells mainly granulocytes (blue arrow) and monocytes (green arrow) in the mucosa were observed in LPS-challenged piglets. Butyrate-treated piglets showed dramatically improved parameters, which were directly reflected by histological scores. Compared with the LPS group, histological scores in the BT-LPS group were significantly decreased (*p* < 0.05), but significantly higher than those in the CON group (*p* < 0.05; Figure 1C).

**Figure 2:**
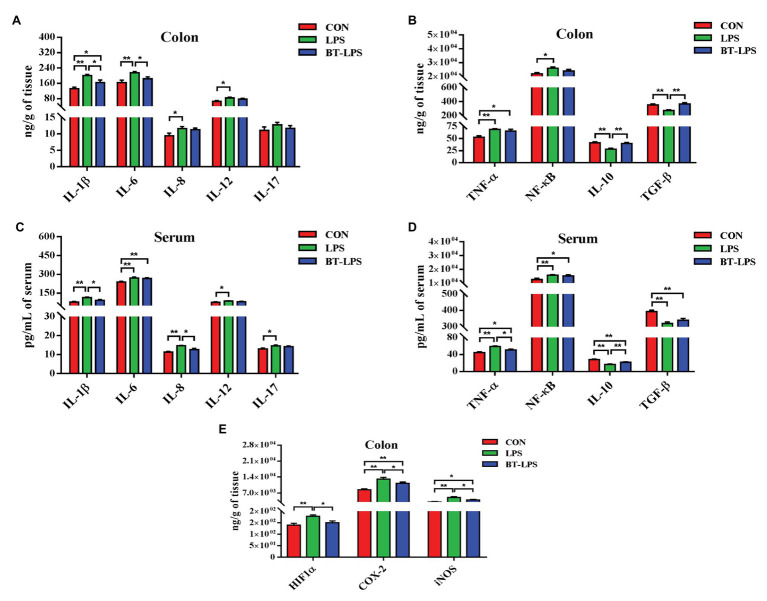
Dietary supplementation with butyrate mitigated colitis of weaned piglets caused by LPS. **(A,B)** Colonic pro-inflammation-associated parameters and anti-inflammation parameters, respectively. **(C,D)** Serum pro-inflammation-associated parameters and anti-inflammation parameters of weaned piglets, respectively. **(E)** Transcriptional regulatory factor (HIF1α) and its targeted protein levels (COX-2 and iNOS) in colonic tissue. All values are expressed as the mean ± SEM (*n* = 6). ^*^p < 0.05 and ^**^p < 0.01.

Compared with the CON group, the LPS group had higher colonic levels of IL-1β, IL-6, IL-8, IL-12, TNF-α, and NF-κB (*p* < 0.05) and lower levels of IL-10 and TGF-β (*p* < 0.05; Figures 2A,B). The BT-LPS group had lower colonic pro-inflammation parameters (IL-1β and IL-6; *p* < 0.05) and higher levels of anti-inflammation parameters (IL-10 and TGF-β; *p* < 0.05) than those in the LPS group, while it had higher levels of IL-1β and TNFα (*p* < 0.05) than those in the CON group. Meanwhile, a similar change in cytokines was found in the serum (Figures 2C,D). In comparison with the CON group, LPS challenge significantly increased colonic HIF1α level (*p* < 0.05), which was significantly decreased with butyrate treatment (*p* < 0.05; Figures 2E). COX-2 and iNOS levels in the LPS and BT-LPS groups were higher than those in the CON group (*p* < 0.05), while levels in the BT-LPS group were lower than those in the LPS group (*p* < 0.05).

### Butyrate Supplementation Increased Concentrations of Butyric Acid in the Colon and Serum, and Induced a Shift of Microbiota

**Figure 3:**
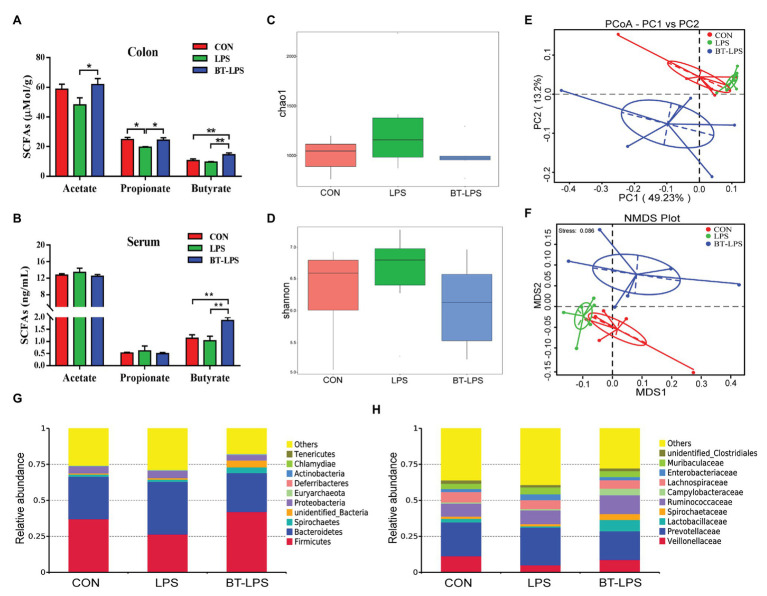
Butyrate supplementation increased concentrations of butyric acid in colon and serum and induced a shift of microbiota. **(A,B)** Concentrations of short-chain fatty acids (SCFAs) in colon and serum. **(C,D)** Bacterial richness and diversity in the CON, LPS, and BT-LPS groups were estimated using the Chao 1 value and Shannon index, respectively. **(E,F)** Principal coordinate analysis and nonmetric multidimensional scaling analysis based on weighted Unifrac distance displayed a separation between the BT-LPS group to the CON and LPS groups. **(G,H)** Distribution of colonic bacteria at the phylum and family levels. All values are expressed as the mean ± SEM (*n* = 6). ^*^p < 0.05 and ^**^p < 0.01.

Higher colonic and serum concentrations of butyric acid were detected in the BT-LPS group compared with the CON and LPS groups (*p* < 0.05; Figures 3A,B). However, butyrate supplementation significantly increased concentrations of colonic acetic and propionic acids in comparison with the LPS group (*p* < 0.05), but no difference was observed between the BT-LPS and CON groups (*p* > 0.05). Butyrate supplementation and LPS challenge did not significantly affect the alpha diversity of the bacterial community including richness (Chao 1) and diversity (Shannon; *p* > 0.05; Figures 3C,D). Weighted PCoA and NMDS plot analysis showed that the colonic microbial communities were well-separated among groups (Figures 3E,F).

Compared with the CON group, the LPS challenge decreased the relative abundance of *Firmicutes* by 10.54% and increased the relative abundance of *Bacteroidetes* and *Proteobacteria* by 7.04 and 0.33%, respectively (Figure 3G). In comparison with the LPS group, butyrate supplementation significantly increased the relative abundance of *Firmicutes* by 15.6% (*p* < 0.05) and decreased the relative abundance of *Bacteroidetes* and *Proteobacteria* by 9.55 and 1.31%, respectively (*p* > 0.05). At family level, the relative abundance of *Lactobacillaceae* in the BT-LPS group was 7.97% higher than the 0.89% in the LPS group (*p* < 0.05), even higher than 2.59% in the CON group (*p* > 0.05), and *Enterobacteriaceae* in the LPS group was 4.09% higher than the 2.07% in the CON group and 2.16% in the BT-LPS group (*p* > 0.05), respectively (Figure 3H).

**Figure 4:**
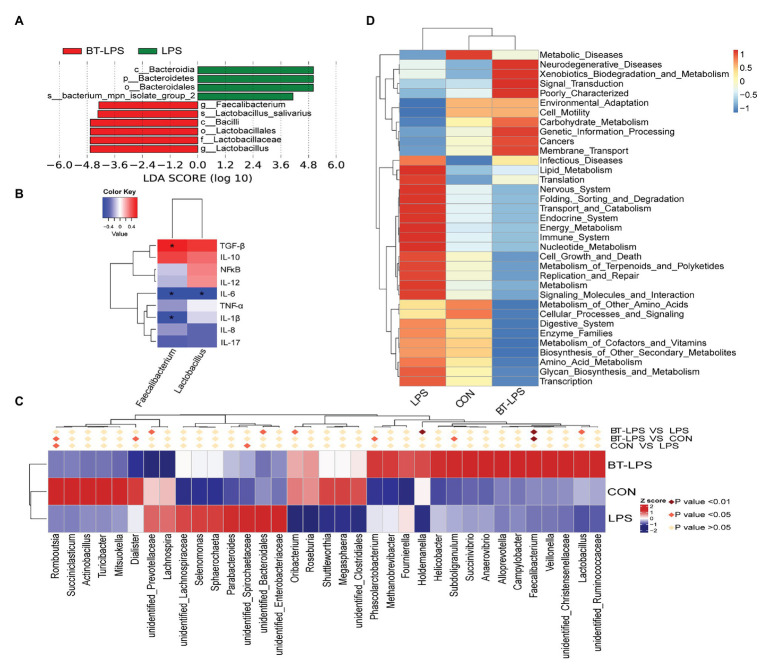
Statistical analysis of difference on microbiota and Phylogenetic investigation of communities by reconstruction of unobserved states (PICRUSt) analysis. **(A)** Enrichment of taxa based on linear discriminant analysis effect size (LEfSe) analysis revealed significant differences of microbial community among the three groups. Bacterial taxa with logarithmic LDA score > 4 were selected as biomarker taxa. (p, phylum level; c, class level; o, order level; f, family level; g, genus level; s, species level). **(B)** Spearman correlation analysis between the relative abundances of beneficial bacteria on the genus level and colonic cytokines. **(C)** Heatmap of the relative abundance in the top 35 genera in the colon. Metastat analysis was performed to test significant differences of relative abundance of genera, with light pink diamond indicating *p* > 0.05, deep pink diamond indicating *p* < 0.05, and dark pink diamond indicating *p* < 0.01, between each two groups. **(D)** Top 35 predictive metabolism pathways on KEGG level II based on PICRUSt analysis. ^*^p < 0.05.

Linear discriminant analysis effect size analysis indicated that *Bacteroidetes* was dominant with all the differences in the relative abundance belonging to this phylum in the LPS group (Figure 4A). In contrast, in the BT-LPS group, *Lactobacillus* and *Faecalibacterium* belonging to *Lactobacillales* and *Clostridiales*, respectively, constituted the dominant bacteria. Of the 35 most dominant genera, the relative abundance of *Faecalibacterium* in the BT-LPS group was significantly higher than that in the CON and LPS groups (*p* < 0.01), and the relative abundance of *Lactobacillus* was higher than that in the LPS group (*p* < 0.05; Figure 4C). Spearman correlation analysis showed that *Lactobacillus* and *Faecalibacterium* had significant negative associations with IL-6 (*p* < 0.05), and *Faecalibacterium* significantly positively correlated with TGF-β and negatively correlated with IL-1β (*p* < 0.05; Figure 4B). The PICRUSt analysis suggested that immune function was modified in bacterial functionalities on KEGG pathway-level II, and colon bacteria in butyrate-fed piglets were thought to provide a lower risk in both infectious diseases and immune system (Figure 4D).

### Butyrate Altered the Energy Metabolism Profile in the Gut–Liver Axis

**Figure 5:**
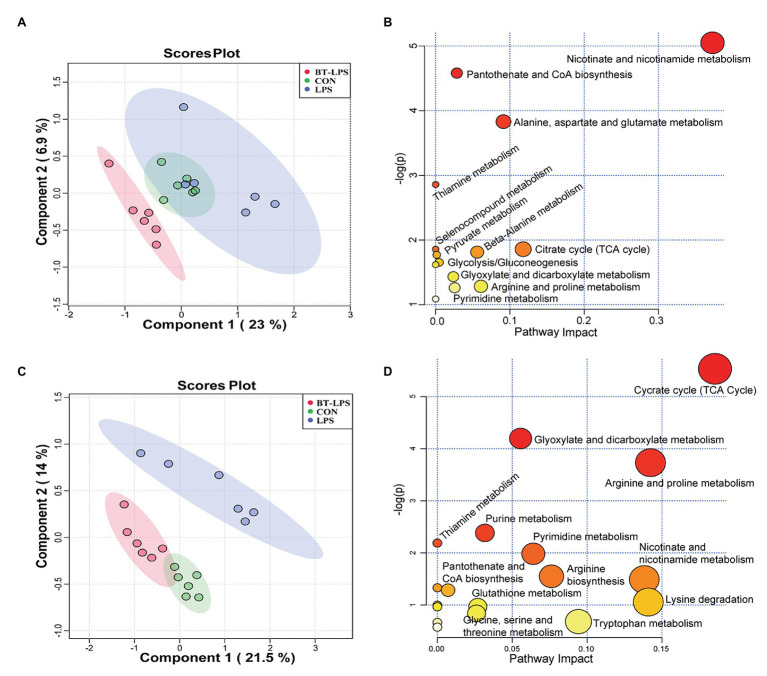
Butyrate supplementation altered the energy metabolism profile in the gut–liver axis. **(A,C)** Partial least squares–discriminant analysis plots, in the colon and liver, show that hydrophilic metabolome in the gut–liver was affected by LPS challenge and butyrate administration. **(B,D)** In the colon and liver, overview of pathway analysis of significant metabolites. Each circle displays one matched pathway, with the color and size of each circle based on the *p*-value and the pathway impact value, respectively.

A total of 169 hydrophilic metabolic compounds were determined using an in-house built mass spectrometry-based targeted metabolomics approach. PLS–DA analysis showed an obvious separation among different treatments in the colon (Figure 5A) and liver (Figure 5C). Fourteen pathways were involved in the colon, among which five pathways contributed most, including nicotinate and nicotinamide metabolism, pantothenate and CoA biosynthesis, alanine, aspartate, and glutamate metabolism, and thiamine metabolism and citrate (TCA) cycle (Figure 5B). In addition, 18 pathways were involved in the liver, among which 10 pathways contributed the most, namely TCA cycle, glyoxylate and dicarboxylate metabolism, arginine and proline metabolism, purine metabolism, pyrimidine metabolism, thiamine metabolism, nicotinate and nicotinamide metabolism, arginine biosynthesis, lysine degradation, and tryptophan metabolism (Figure 5D).

**Figure 6:**
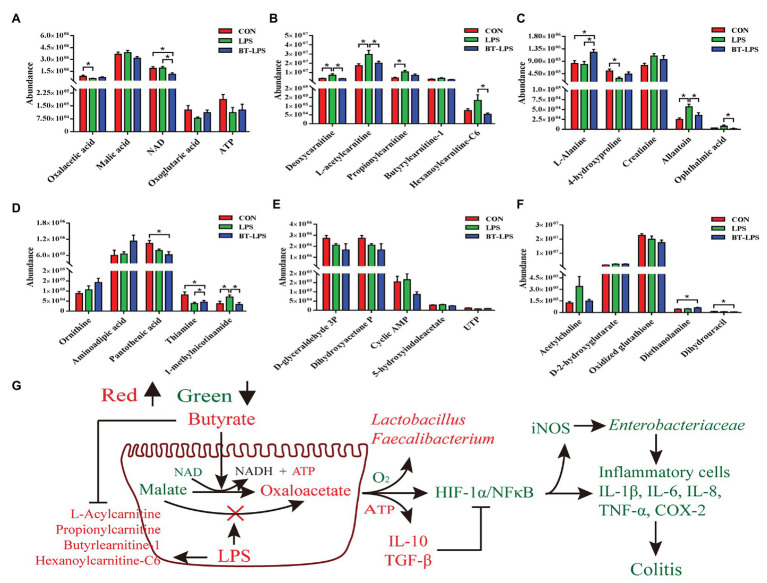
Hydrophilic metabolites in the colon were altered by butyrate supplementation. **(A–F)** Colonic hydrophilic metabolites with significant difference (*p* < 0.05) and with difference for tendency (0.05 < *p* < 0.1) between groups. **(G)** The integrated immune indexes, 16S rRNA gene sequencing, and targeted metabolome revealed that butyrate supplementation mitigates weanling piglets from LPS-induced colitis *via* regulating microbiota and energy metabolism of the colon. The red and green colors represent increased and decreased immune parameters for microbes and metabolites, respectively, in the BT-LPS group compared with those in the LPS or CON groups. All values are expressed as the mean ± SEM (*n* = 6). ^*^p < 0.05.

Correspondingly, in the colon, Duncan’s multiple-range test and LSD *post hoc* tests indicated that 15 metabolites were significantly affected (*p* < 0.05) and 17 metabolites trended to being affected by butyrate supplementation (0.05 < *p* < 0.01; Figures 6A–F). Compared with the CON group, LPS challenge significantly decreased colonic abundance of oxaloacetic acid and 4-hydroxyproline (*p* < 0.05), but increased the abundance of 1-methylnicotinamide and allantoin (*p* < 0.05). Butyrate administration significantly increased the abundance of l-alanine, thiamine, and diethanolamine (*p* < 0.05), but significantly decreased the abundance of NAD, 1-methylnicotinamide, allantoin, and ophthalmic acid (*p* < 0.05), in comparison with the LPS group. LPS challenge significantly increased the abundance of deoxycarnitine, l-acetylcarnitine, propionylcarnitine, and hexanoylcarnitine-C6 (*p* < 0.05) compared with the CON group, butyrate treatment significantly decreased the abundance of deoxycarnitine, l-acetylcarnitine, and hexanoylcarnitine-C6 (*p* < 0.05), and tended to decrease the abundance of propionylcarnitine and butyrylcarnitine-1 (0.05 < *p* < 0.1), in comparison with the LPS group. These acylcarnitines showed no significant difference between the BT-LPS and CON groups (*p* > 0.05). Butyrate supplementation tended to protect against an increase of malic acid and a decrease of adenosine triphosphate (ATP) and oxoglutaric acid induced by LPS (0.05 < *p* < 0.1; Figure 6G).

**Figure 7:**
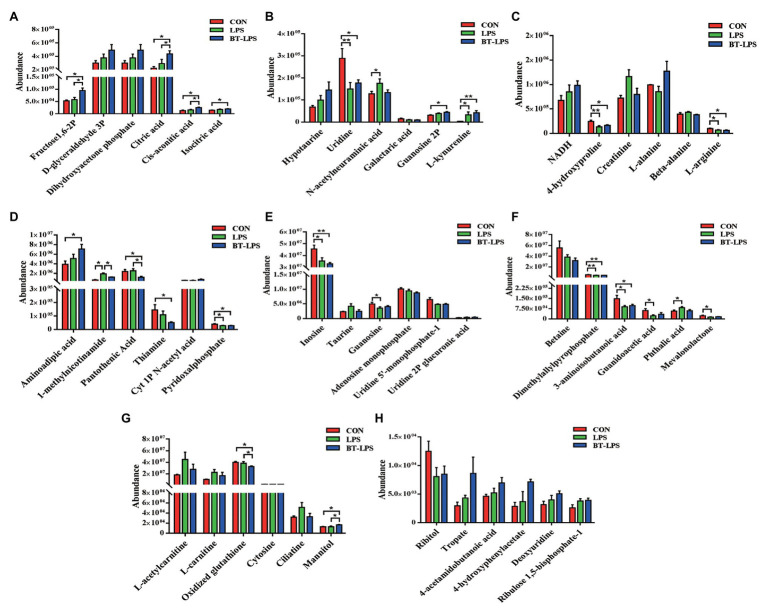
Hydrophilic metabolites in the liver were altered by butyrate supplementation. **(A–H)** Hydrophilic metabolites with significant difference and with difference for tendency (0.05 < *p* < 0.1) between groups. All values are expressed as the mean ± SEM (*n* = 6). ^*^p < 0.05 and ^**^p < 0.01.

In the liver, 28 metabolic compounds were significantly affected by LPS challenge and butyrate administration (*p* < 0.05), and another 24 species were trend affected (0.05 < *p* < 0.1; Figure 7). Compared with the CON or LPS groups, butyrate supplementation enhanced the TCA cycle, reflected by significantly increasing liver levels of fructose-1,6-bisphosphate, citric acid, *cis*-aconitic acid, and isocitric acid (*p* < 0.05), and tended to increase NADH (0.05 < *p* < 0.1; Figures 7A,C). Butyrate administration altered amino acid metabolism, with decreasing levels of 4-hydroxyproline, l-arginine (*p* < 0.05), and increasing levels of l-alanine (0.05 < *p* < 0.1) and l-kynurenine (*p* < 0.05) in comparison with the CON or LPS group (Figures 7B,C). The levels of pyridoxal phosphate and thiamine in the BT-LPS group were lower than those in the CON group (*p* < 0.05), and the levels of 1-methylnicotinamide and pantothenic acid were lower than those in the LPS group (*p* < 0.05; Figure 7D). Consistent with the results in the colon, LPS challenge increased levels of l-acetylcarnitine and l-carnitine (0.05 < *p* < 0.1), which were reduced by butyrate treatment (Figure 7G). In comparison with the CON group, LPS challenge significantly decreased 3-aminoisobutanoic acid, guanidoacetic acid, and mevalonolactone, and significantly increased phthalic acid (*p* < 0.05; Figure 7F). Butyrate administration reversed those LPS-induced changes to some extent (*p* > 0.05).

### Correlation Analysis Between the Colonic Metabolites and Cytokines

**Figure 8:**
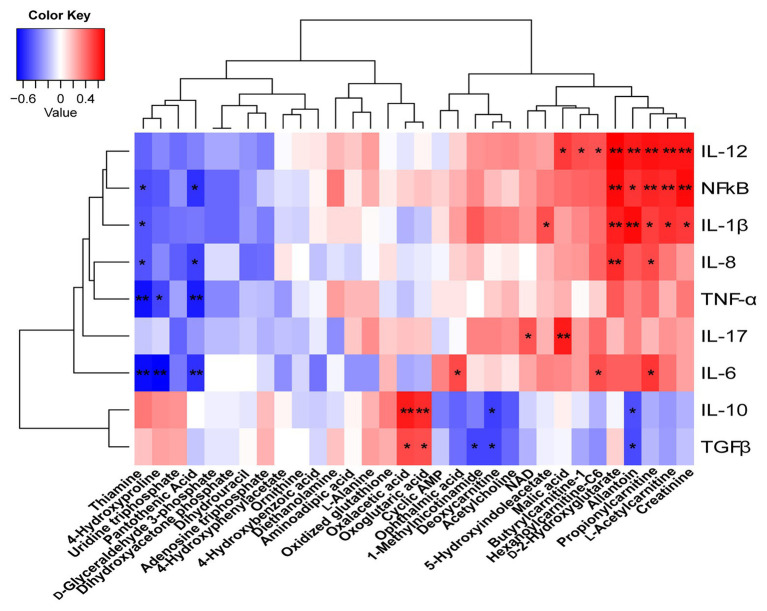
Correlations between the colonic cytokines and metabolites. Red represents a positive correlation and blue represents a negative correlation. ^*^p < 0.05 and ^**^p < 0.01.

To understand how butyrate impacts host immune homeostasis through altering metabolism in the gut–liver axis, a Spearman’s correlation matrix was generated to explore these relationships (Figure 8). Between colonic cytokines and metabolites, IL-1β, IL-12, NF-κB had a positive correlation with creatinine, l-acetylcarnitine, propionylcarnitine, allantoin, and d-2-hydroxyglutarate (*p* < 0.05), and IL-8 had a positive correlation with propionylcarnitine and d-2-hydroxyglutarate (*p* < 0.05). Both oxaloacetic and oxoglutaric acids were positively correlated with IL-10 and TGF-β (*p* < 0.05), yet IL-6, TNF-α, and NF-κB were negatively correlated with thiamine, pantothenic acid, and 4-hydroxyproline, respectively (*p* < 0.05). IL-6 had a positive correlation with propionylcarnitine, hexanoylcarnitine-C6, and ophthalmic acid (*p* < 0.05). IL-10 and TGF-β were negatively correlated with deoxycarnitine and allantoin (*p* < 0.05).

## Discussion

Lipopolysaccharide challenge damages the colonic morphology of piglets, reflected by the lower epithelial quality, and a significant increase in the ratio of crypt depth to tissue thickness (Geng et al., 2018). This is in accordance with the present results. This pathological recovery was enhanced by butyrate supplementation, which was directly reflected by the improved histological scores in epithelial damage, infiltration of inflammatory cells, and edema in the submucosa. A similar result was observed by Fachi et al. (2019), who found an improvement in colon histological scores in butyrate-treated mice. Because cytokines are involved in the regulation of intestinal homeostasis (Huang et al., 2015), we further observed the effects of butyrate on the immune response in colonic tissue and serum. Consistent with the histological observation, the present study indicated butyrate diminished the increase of pro-inflammatory cytokines, such as IL-1β, IL-6, TNFα, IL-8, and IL-12 caused by LPS. This agrees with the previous studies. Parenteral supplemented with sodium butyrate in a manner of intravenous infusion also significantly decreased the gene expression of colonic proinflammatory cytokines IL-6, IL-18, IL-12p40, and TNF-α, but increased the expression of intestinal development-related genes *ZO-1*, occluding, and *EGF* (Chen et al., 2018b). Moreover, butyrate ameliorated *Clostridium difficile*-induced colonic inflammatory responses as indicated by significantly decreased inflammatory cytokines such as IL-1β, IL-6, and CXCL-1 (Fachi et al., 2019). Indeed, as reported, butyrate decreased the expression of proinflammatory cytokines in patients with IBD (Segain, 2000). Thus, these indicate that butyrate could decrease the inflammatory response, and then improve the histological status.

In the present study, except for increased butyrate concentrations, dietary supplementation with butyrate increased colonic acetate and propionate concentrations in comparison with the LPS group, which were associated with a shift of microbial community by butyrate administration. *Phascolarctobacterium* and *Veillonella* were reported to ferment carbohydrates into propionate by the propanediol pathway (Scott et al., 2006). *Ruminococcus* and *Mitstuokella* were reported as acetate-producing bacteria (Koh et al., 2016; Vargas et al., 2017). In the current study, these bacterial levels in the BT-LPS or CON groups were higher than those in the LPS group, which was consistent with the increased production of propionic acid and acetic acid. *Megaphaera* increased in the CON and BT-LPS groups, which could convert lactate to acetate *via* the methylmalonyl-CoA pathway, and further utilize both acetate and lactate to synthesize butyrate by butyryl-CoA:acetate-CoA transferase (Zhang et al., 2018a). Coupled with the present result, the relative abundance of *Lactobacillus* was increased in the BT-LPS group. These well explained a change in SCFAs, and indicated that there exists a shift and an inner conversion of microbiota under different treatments.

A previous study showed that infusion with sodium butyrate tended to increase the proportion of colonic *Firmicutes*, *Spirochaetae*, and unclassified *Clostridiales* but decrease the relative abundance of *Bacteroidetes* (Chen et al., 2018b). Similarly, dietary supplementation with sodium butyrate increased the relative abundance of *Clostridiaceae*, *Lachnospiraceae*, and *Ruminococcaceae* in the colon of weaned piglets (Huang et al., 2015). This is basically consistent with the results of the current study. Concretely, dietary supplementation with butyrate significantly increased the relative abundance of *Lactobacillus* and *Faecalibacterium*. *Lactobacillus* has been used as an indicator of intestinal health. As reported, a decrease of *Lactobacillus* level was found in IBD patients compared with that in the control, while the *Enterobacteriaceae* level was increased (Ailing et al., 2020). In a *Staphylococcus aureus*-infected model, *Lactobacillus* administration could efficiently decrease the number of *Staphylococcus aureus*, suppress the inflammatory cytokines TNF-α and IL-6, and repair damage to the intestinal barrier (Liu et al., 2020). This was mainly reflected by a change of intestinal villi length, and up-regulated expressions of *ZO-1* and occluding. Moreover, *Lactobacillus* treatment alleviated colonic hemorrhage in DSS-induced colitis mice by protecting from the destructive damage of goblet cells and preserving the integrity of the epithelial structure in the colon, with significantly increasing ZO-1 expression in protein levels (Shin et al., 2020). This contributed to a lower disease activity score of DSS-induced colitis mice, which coincided with the results of the present study. Hence, *Lactobacillus* plays a pivotal role in decreasing inflammatory cytokines, promoting gut barrier recovery and thereby alle*via*ting colitis.

Butyrate-producing *Faecalibacterium* is a common anaerobe and considered as a promising next-generation probiotic, the absence of which is closely connected with IBD (Lukovac et al., 2014). *Faecalibacterium* seems to be associated with immunity homeostasis. As reported, *Faecalibacterium* supplementation prevented an acute trinitrobenzene-sulfonic acid-induced increase in the proinflammatory cytokines such as IL-8 and IL-12, but presented an anti-inflammatory response by increasing IL-10 secretion (Sokol et al., 2008). In addition, a previous study showed that *Faecalibacterium* helped control hyperpermeability in a model of inflammatory bowel disease (Martín et al., 2015). Prophylactic treatment with *Faecalibacterium* also decreased radiation-induced colonic epithelial barrier rupture at day 3, and then led to a decreased area of mucosal ulceration at day 7 (Lapiere et al., 2020). Moreover, preclinical experiments performed in animal models of inflammatory bowel disease have demonstrated that *Faecalibacterium* treatment reduced colonic damage and increased survival of animals (Sokol et al., 2008; Huang et al., 2016; Lapiere et al., 2020). Therefore, *Faecalibacterium* also functions to maintain intestinal health. These findings appear to coincide with those of the current study. Combined with PICRUSt and correlation analyses, this indicated that dietary supplementation with butyrate improved severe colitis partly by accelerating the prevalence of *Lactobacillus* and *Faecalibacterium*.

Colonic dysbiosis was commonly associated with an increase in the relative abundance of facultative anaerobic bacteria, which was found in individuals suffering from IBD (Morgan et al., 2012). This agrees with the present results that LPS increased *Enterobacteriaceae*, which we hypothesize will be aggravated with long-term treatment of LPS. *Enterobacteriaceae* as members of facultative anaerobic bacteria interfered with host nutrition by metabolizing fermentation products to carbon dioxide (Litvak et al., 2018). Butyrate supplementation effectively suppressed this facultative anaerobic bacteria proliferation (Fang et al., 2019), which is consistent with the current results. The reason was related to butyrate-changed metabolism of colonocytes. As expected, the present study indicated that butyrate accelerated the process of malic acid conversion to oxaloacetic acid broken by LPS. The potential mechanism was that butyrate administration tended to consume higher oxygen by enhancing oxidative phosphorylation, which contributes to maintaining an anaerobic microenvironment for obligated anaerobic communities (Litvak et al., 2018; Figure 6G).

Butyrate was imported *via* colonocytes by the transporters MCT1 and SMCT1, and was further metabolized through β-oxidation and the TCA pathway (Allaire et al., 2018). In the present study, butyrate supplementation enhanced the TCA cycle in the gut–liver axis, which was reflected by increasing colonic levels of oxaloacetic and oxoglutaric acids, and increasing liver levels of citric acid, *cis*-aconitic acid, and isocitric acid (Figures 6A, 7A). Meanwhile, the BT-LPS group had a lower NAD level but higher levels of NADH and ATP in the gut–liver axis than the LPS group. Moreover, butyrate supplementation significantly decreased levels of pantothenic acid, thiamine, and 1-methylnicotinamide in the colon and liver, and decreased pyridoxal phosphate level in the liver, compared with the CON or LPS groups, indicating that more of those participated in host metabolism after butyrate addition. As reported, B group vitamins, mostly as a cofactor for several enzymes, catalyzed energy metabolism, which played an important role in the maintenance of balance between the TCA cycle and glycolysis, particularly in the maintenance of the TCA cycle for the generation of ATP (Yoshii et al., 2019). As expected for increased butyrate levels, the present study indicated that colonic acetate concentration was significantly increased in the BT-LPS group, but no significant difference was found in serum between the groups. The probable reason is that acetate was well utilized by the gut wall. Isotopic tracer results indicated the absorbed acetate rapidly converted into aspartate and glutamate, which are hardly exported, and slowly oxidized to carbon dioxide (Vernay et al., 1985). The glutamate, as the preferred respiratory fuel, further energized for the intestines of mammals and enhanced the intestinal barrier and antioxidative functions (Jiao et al., 2015), whose carbon skeleton might be converted into alanine, where it is extensively oxidized (Watford et al., 1979). Alanine, as one of the glucogenic amino acids, further participated in the energy metabolism. In addition, in the present study, propionate concentration had a similar change with acetate, which was consistent with the previous study. Cecal infusion of sodium propionate significantly increased the concentration of propionate in the colon, but no difference was found in serum and liver (Yu et al., 2019). However, sodium propionate infusion increased levels of TCA cycle components, such as malic acid, fructose-6-phosphate, and succinic acid. Moreover, propionate infusion significantly increased the colonic length, suggesting that the propionate was absorbed in the colon as an energy source (Zhang et al., 2019) and transported to the liver for further metabolizing, and thus is present at a low level in the periphery (Sahuri-Arisoylu et al., 2016). There is a possibility that we did not catch the changes of propionate in serum, which might be partly contributed to metabolism in the gut–liver axis.

Amino acids are involved in energy metabolism and other essential life processes of organisms, and any organism injury might cause disorders in amino acid metabolism. In the present study, the LPS challenge caused a depletion of 4-hydroxyproline and l-arginine in the gut–liver axis. As reported, the cooperation of arginine and adenosine could protect tissues exposed to stresses (Jiang et al., 2020). This was in concert with those changes in the BT-LPS group in the present study. Arginine, proline, and alanine are glucogenic amino acids that also contribute to energy metabolism by increasing some intermediates in the pool of the TCA cycle (Galsgaard et al., 2019; Jiang et al., 2020). Combined with a significant or modest increase of fructose-1,6-bisphosphate, d-glyceraldehyde-3-phosphate, and dihydroxyacetone phosphate after LPS challenge, this indicated that the liver might overcome LPS-induced stress by utilizing glucogenic amino acids to synthesize more glucose and to accumulate energy for cellular physiological regulation. However, compared with the other two groups, butyrate supplementation significantly increased the level of alanine in the gut–liver axis, which converted from pyruvate. This might have resulted from the butyrate-enhanced glucose–alanine cycle. In turn, positional isotopomer NMR tracer analysis indicated that the glucose–alanine cycle was enhanced by alanine infusion and contributed to hepatic mitochondrial oxidation and produced more ATP in humans (Petersen et al., 2019). Thus, those were the responses to butyrate supplementation to overcome stressors, such as LPS challenge.

Hydrophilic metabolites seem to be associated with inflammation response, such as accumulation of citrate and succinate when the TCA cycle is disrupted (Jha et al., 2015). Here, we propose that energy metabolism in the gut–liver axis plays an important role in maintaining immunity homeostasis. SCFAs promote T-regulatory cell differentiation and production of TGF-β, and resting immune cells switch fuel usage from glucose to fatty acids and ketones (Trapecar et al., 2020). In addition, quiescent and T-regulatory cells mainly use the TCA cycle for energy generation (Buck et al., 2017). Those were consistent with the present results and explained well that butyrate supplementation did promote TGF-β and IL-10 production. It is known that T-regulatory cell production of IL-10 has been considered as a self-limiting mechanism to prevent an exaggerated T-cell response, and to regulate intestinal homeostasis and protect against IBD (Furusawa et al., 2013). TGF-β has a positive effect on epithelial recovery and the amelioration of the inflammatory process (Geng et al., 2018), while TGF-β deficiency accelerated dysfunction of the immune system and resulted in severe IBD (Kotlarz et al., 2018). These indicated that butyrate administration enhancing the TCA cycle in the gut–liver axis contributed to immunity homeostasis.

Intriguingly, in the current study, LPS challenge resulted in increases in levels of acylcarnitines in the colon and liver, which were reduced by butyrate administration (Figures 6B, 7G). As reported, LPS triggered an increase in fatty acid synthesis in macrophages (Feingold et al., 2012). Fatty acid synthesis was observed to be upregulated during TLR-mediated dendritic cell activation (Everts et al., 2014). In addition, fatty acid synthesis was also found to be necessary for cell proliferation after the activation of T and B cells *via* antigen receptors (Chen et al., 1975; Dufort et al., 2014). Accompanied by the present results in correlation analysis and pathway analysis, those of acylcarnitines were significantly positively associated with proinflammatory factors in the colon (Figure 8), as well as connected with CoA biosynthesis. These results indicated that butyrate supplementation suppressed lipid acid metabolism in the gut–liver axis, which was contributed to immunity homeostasis. Creatinine was seen as a marker in conventional clinical renal damage, and some patients with chronic kidney disease had an increase (Aparicio-Trejo et al., 2020). In agreement with the present results, LPS challenge caused an increased creatinine level in the gut–liver axis and was associated with a proinflammatory response. Dietary intervention with butyrate reversed this inflammatory response status. Those results and evidence were fully explained by the present result that lower infiltration of inflammatory cells was observed in the BT-LPS group.

HIF1α activated the master regulator of the inflammatory response of NF-κB and then resulted in inflammation (Scortegagna et al., 2008), producing proinflammatory cytokines such as IL-1β, IL-6, TNF-α, and iNOS (Fan et al., 2019). The potential mechanism of HIF1α activating NF-κB was through two independent pathways involved in IκB phosphorylation and ERK1/2-mediated phosphorylation of serine 276 on p65 (Scortegagna et al., 2008). The iNOS was reported to promote nitric oxide converting into nitrate and further provided additional electron acceptors for facultative anaerobic bacteria (Winter et al., 2014). Increased availability of nitrate permitted *Enterobacteriaceae* to expand in mouse models of IBD (Hughes et al., 2017). These results are consistent with those of the current study. COX-2 is an inducible key enzyme of inflammatory prostanoids, and its overexpression was correlated with destroying the intestinal barrier (Short et al., 2013). This is consistent with the present study insofar as the protection offered by butyrate against gut barrier-epithelial damage might be associated with a decrease of COX-2. Destroying the TCA cycle can stabilize HIF1α and increase transcription of target genes such as *IL-1β* (Tannahill et al., 2013; Jha et al., 2015; Corcoran and O’Neill, 2016). The current study indicated that butyrate administration enhanced the TCA cycle, which was the reason that the colonic level of HIF1α decreased in the BT-LPS group. However, the mechanism of inactivation of HIF1α resulting from metabolism in the gut–liver axis was hitherto unclear. Hence, more research is needed to clarify the role played by HIF1α between the metabolism of the gut–liver axis and immunity homeostasis.

## Conclusion

**Figure 9:**
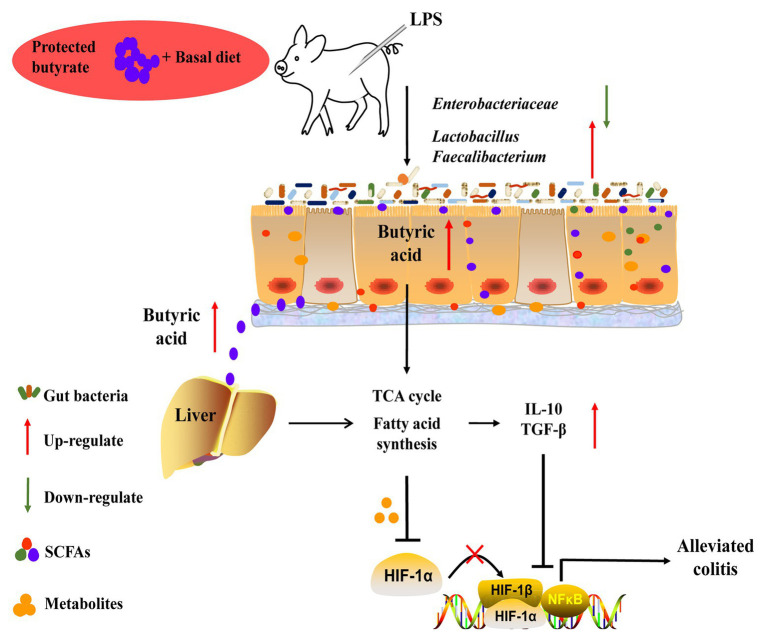
Schematic representation for the improvement of butyrate in colitis in LPS-challenged piglets. Butyrate protected against colitis by changing energy metabolism in the gut–liver axis, assisting the prevalence of *Lactobacillus* and *Faecalibacterium*, and enhancing colonic immunity homeostasis.

The present study showed that protected butyrate reshaping of colonic microbiota and energy metabolism in the gut–liver axis was protective against colitis (Figure 9). This protective effect resulted from the improved immunity homeostasis and was closely connected with the prevalence of colonic *Lactobacillus* and *Faecalibacterium*, elevation of the TCA cycle, and suppression of lipid acids’ synthesis in the gut–liver axis. During this process, HIF1α might play an important role between metabolism in the gut–liver axis and immunity homeostasis. Because our understanding of the molecular details and signaling pathways resulted in reshaping colonic microbiota and energy metabolism in the gut–liver axis, targeted restoring of butyrate levels in the colon may be an alternative form of therapy for colitis.

## Data Availability Statement

The datasets generated for this study can be found in the NCBI sequence read archive, accession number PRJNA648691.

## Ethics Statement

The animal study was reviewed and approved by Institutional Animal Care and Use Committee of the Institute of Animal Science of the Chinese Academy of Agricultural Sciences.

## Author Contributions

YH, QZ, JZ, and FL designed the experiments. YH, QZ, YL, and KZ conducted the experiments and collected the samples. YH, YL, and KZ performed the analysis of samples. YH, QZ, and CT analyzed the data. YH, JZ, and FL wrote and revised the manuscript. All authors contributed to the article and approved the submitted version.

### Conflict of Interest

The authors declare that the research was conducted in the absence of any commercial or financial relationships that could be construed as a potential conflict of interest.

## Footnotes

^1^http://www.drive5.com/usearch/manual/uchime_algo.html

^2^http://www.arb-silva.de/

^3^http://www.mothur.org/wiki/Chao

^4^http://www.mothur.org/wiki/Shannon

## Abbreviations

ATP: Adenosine triphosphate
BW: Body weight
COX-2: Cyclooxygenase 2
DSS: Dextran sulfate sodium
HIF1α: Hypoxia-inducible factor 1α
IBD: Inflammatory bowel disorders
iNOS: Inducible nitric oxide synthase
IL: Interleukin
LPS: Lipopolysaccharide
MD-2: Myeloid differential protein-2
MyD88: Myeloid differentiation factor 88
NF-κB: Nuclear factor κB
OTUs: Operational taxonomic units
SCFAs: Short-chain fatty acids
TGF-β: Transcription growth factor β
TLR: Toll-like receptor
TNF-α: Tumor necrosis factor
